# Comparison Between Levodopa-Carbidopa Intestinal Gel Infusion and Subthalamic Nucleus Deep-Brain Stimulation for Advanced Parkinson's Disease: A Systematic Review and Meta-Analysis

**DOI:** 10.3389/fneur.2019.00934

**Published:** 2019-08-27

**Authors:** Xiao Dong Liu, Yi Bao, Guang jian Liu

**Affiliations:** Department of Neurology, Taihe Hospital, Hubei University of Medicine, Shiyan, China

**Keywords:** Parkinson's disease, Levodopa-Carbidopa intestinal gel infusion, deep-brain stimulation, comparison, meta-analysis

## Abstract

**Background:** Currently, some advanced treatments such as Levodopa-Carbidopa intestinal gel infusion (LCIG), deep-brain stimulation (DBS), and subcutaneous apomorphine infusion have become alternative strategies for advanced Parkinson's disease (PD). However, which treatment is better for individual patients remains unclear. This review aims to compare therapeutic effects of motor and/or non-motor symptoms of advanced PD patients between LCIG and DBS.

**Methods:** We manually searched electronic databases (PubMed, Embase, Cochrane Library) and reference lists of included articles published until April 04, 2019 using related terms, without language restriction. We included case-controlled cohort studies and randomized-controlled trials, which directly compared differences between LCIG and DBS. The Newcastle-Ottawa scale (NOS), proposed by the Cochrane Collaboration, was utilized to assess the quality of the included studies. Two investigators independently extracted data from each trial. Pooled standard-mean differences (SMDs) and relative risks (RRs) with 95% confidence intervals (CIs) were calculated by meta-analysis. Outcomes were grouped according to the part III and part IV of the Unified Parkinson Disease Rating Scale (UPDRS) and adverse events. We also descriptively reviewed some data, which were unavailable for statistical analysis.

**Results:** This review included five cohort trials of 257 patients for meta-analysis. There were no significant differences between LCIG and subthalamic nucleus deep-brain stimulation (STN-DBS) on UPDRS-III and adverse events comparisons: UPDRS-III (pooled SMDs = 0.200, 95% CI: −0.126–0.527, *P* = 0.230), total adverse events (pooled RRs = 1.279, 95% CI: 0.983–1.664, *P* = 0.067), serious adverse events (pooled RRs = 1.539, 95% CI: 0.664–3.566, *P* = 0.315). Notably, the improvement of UPDRS-IV was more significant in STN-DBS groups: pooled SMDs = 0.857, 95% CI: 0.130–1.584, *P* = 0.021. However, the heterogeneity was moderate for UPDRS-IV (*I*^2^ = 73.8%).

**Conclusion:** LCIG has comparable effects to STN-DBS on motor function for advanced PD, with acceptable tolerability. More large, well-designed trials are needed to assess the comparability of LCIG and STN-DBS in the future.

## Introduction

Levodopa is currently one of the most effective drugs for Parkinson's disease (PD) (1). However, long-term treatment of levodopa is frequently associated with complications such as motor fluctuation (2) and dyskinesia (3). As a result, some advanced treatments such as Levodopa-Carbidopa intestinal gel infusion (LCIG) and deep-brain stimulation (DBS) have emerged as alternative strategies for treating PD. However, which treatment is better for advanced PD patients remains unclear. Although some advanced PD patients could benefit from any one of these therapies, it is still important to determine whether there is a better PD treatment for each patient. The adverse effects brought about by PD are severe (4), and there is also a heavy economic burden from PD-specific treatment and care (5). Moreover, once one of these advanced PD therapies is initiated, it may induce irreversible harm to the patient, which cannot be solved by alternative methods (6). Therefore, cautious and rational clinical decisions for both patients and physicians are necessary for routine medical treatments.

Currently, many clinical trials had been carried out to investigate the therapeutic value of these advanced treatments for PD. Regarding LCIG, for example, several randomized-controlled trials (RCTs) (7–9) have indicated that LCIG is effective at improving motor fluctuation, dyskinesia, some non-motor symptoms, and overall quality of life. Similar effects have been affirmed by some clinical trials with regard to DBS (10, 11). However, to our knowledge, relevant trials engaged in comparison between these therapeutic methods have been limited. Hence, the aim of this study was to compare the clinical effects of LCIG and DBS on motor and/or non-motor symptoms using a meta-analysis and a systematical review of the relevant literature. Although the results of this study may not be sufficient for guiding future clinical decisions, they may be helpful in estimating the potential value of carrying out further related research in the future.

## Methods

This systematic review and meta-analysis followed the guidelines for the design, performance, and reporting for meta-analyses of observational studies published by the Meta-Analysis of Observational Studies in Epidemiology (MOOSE) group (12). Since published papers introduced the data in our study, there were no ethical issues involved.

### Data Sources and Searches

Two investigators independently searched PubMed (up to April 04, 2019), Embase (up to April 04, 2019) and the Cochrane Library (Issue 12, April 04, 2019) to acquire all related trials. We used Medical Subject Heading terms (MeSH terms) combined with free texts as the following searching terms: “Parkinson Disease,” “Parkinson's disease,” “parkinsonism” and “PD” for participants; “Levodopa-Carbidopa,” “levodopa/carbidopa,” “intestinal gel,” “gel infusion,” “Duodenal levodopa infusion,” “carbidopa plus levodopa,” “LCIG,” “infusion,” “duodopa” for intervention; “Brain Stimulations, Deep,” “Deep Brain Stimulations,” “Electrical Stimulation of the Brain,” “DBS,” “Deep Brain Stimulation,” “deep brain stimulator,” and “brain depth stimulation” for comparable intervention. There were no language restrictions for searching. In addition, we manually examined the reference lists of all included articles to identify potential eligible trials (see the Supplementary Table 1).

### Study Selection

For study selection, we designed several inclusion/exclusion criteria to acquire eligible trials that could more comprehensively reflect “real world” clinical practices. Firstly, the study design was confined to clinical trials. In another word, only case-controlled, cohort or randomized-controlled trials (RCTs) were eligible for our study. Next, we only included studies reporting direct comparison between LCIG and DBS. Thirdly, we excluded reviews, editorials, letters, case series, case reports, and conference proceedings. Fourthly, the inclusion criteria for all patients should be clear in included studies. Specifically, there must be unified diagnostic criteria or definition for enrolled patients in each study. Fifthly, the patients of included trials couldn't receive any of these advanced therapies before studying (for example, participants who had received DBS before LCIG were ineligible). In addition, they couldn't switch or withdraw these treatments either, once the enrollment was initiated. Finally, we also excluded studies that provided inappropriate analyses leading to potentially high bias from confounding variables.

### Quality Assessment

As all eligible publications were cohort trials, we assessed the methodological quality of these studies according to the Newcastle–Ottawa scale (NOS) recommended by the Cochrane Non-Randomized Studies Methods Working Group (www.cochrane.org) (13). NOS includes the following three subscales: selection, comparability, and outcome. The studies were allocated stars based on specific criteria adjusted by our review. The modified form of NOS is listed in Table 1.

**Table 1:** Newcastle-Ottawa quality assessment scale for cohort studies (modified).

**Selection**
1) Representativeness of the exposed cohort
a) Truly representative of PD patients in the community^*^
b) Somewhat representative of PD patients in the community^*^
c) Didn't select representative PD group of patients
d) No description of the derivation of the cohort
2) Selection of the non-exposed cohort
a) Drawn from a selected PD cohort as the exposed cohort^*^
b) Drawn from a different source
c) No description of the derivation of the non-exposed cohort
3) Ascertainment of exposure
a) Secure record (e.g., surgical records)^*^
b) Validated measure (e.g., structured interview)^*^
c) Written self-report (e.g., diaries)
d) No description
4) Demonstration that any advanced treatment (e.g., DBS) was not present at start of study
a) Yes^*^
b) No
**Comparability**
1) Comparability of cohorts on the basis of the design or analysis
a) Study controls for adequately for age or gender^*^
b) Study controls for disease characteristics as additional factor^*^
**Outcome**
1) Assessment of neurological function or neuropsychological function or adverse event (e.g., dyskinesia)
a) Independent blind assessment with clinical criteria^*^
b) Record linkage^*^
c) Self-report or non-blinded assessment
d) No description (e.g., for blindness)
2) Follow-up was long enough (at least 1 year) for initiation of advanced treatment (e.g., DBS) to occur
a) Yes^*^
b) No
3) Adequacy of follow up of cohorts
a) Complete follow-up—all subjects accounted for^*^
b) Subjects lost to follow-up unlikely to introduce bias: >80% follow-up or description provided of those lost^*^
c) Follow up rate <80% and/or no description of those lost
d) No statement

*A study can be awarded a maximum of one star for each numbered item within the Selection and Outcome categories; A maximum of two stars can be given for Comparability. PD, Parkinson's disease; DBS, Deep-brain stimulation*.

* *Quality star allocated if the condition is satisfied*.

### Data Extraction

Two independent investigators checked all eligible articles, extracted available data and entered them in a predefined datasheet. Any discrepancies were resolved by consensus. The extracted data included authors of the study, year of publication, study design, sample size, age range, gender structure, disease duration, Hohen-Yahr stage, baseline levodopa equivalent daily dose (LEDD), follow-up duration, comparable results (for example, Unified Parkinson Disease Rating Scale (UPDRS) score, dyskinesia, and adverse events) and standard-mean differences (SMDs) or relative risks (RRs) with 95% confidence intervals (CIs) for each investigated comparison. All data were extracted from identified articles without further information. In our review, we mainly focused on UPDRS-III and UPDRS-IV as primary endpoints for motor function and motor complication assessment, total adverse events and serious adverse events as secondary endpoints for safety evaluation (see the Supplementary Tables 2, 3).

### Statistical Analysis

The results of varied comparisons were grouped by UPDRS-III, UPDRS-IV, total adverse events and serious adverse events, while some other data that were unavailable for meta-analysis were also reviewed in our study. We introduced SMDs and RRs for pooled results of different trials to assess comparable outcomes. For evaluation of heterogeneity across the various trials, we used the Chi-square test and calculated the *I*^2^ statistic for each analysis. The severity of heterogeneity was divided by the percentage of total variation across studies: 40% for low heterogeneity, 60% for medium heterogeneity, and 75% for high heterogeneity. We ran a fixed-effects model if there was low heterogeneity across varied trials, otherwise, a random-effects model was utilized. The DerSimonian and Laird-Q method and Mantel-Haenszel method were applied for continuous variables and dichotomous variables, respectively. In addition, we used Galbraith plots to visually check the potential trials as important sources of overall heterogeneity and then conducted a sensitivity analysis by removing the selected trials. Finally, we evaluated the publication bias according to the Begg's test and Egger's method. Stata Statistical Software version SE 12.0 (Stata Corp. LP, College Station, TX, USA) was used for all analyses.

## Results

### Selection of Studies

**Figure 1:**
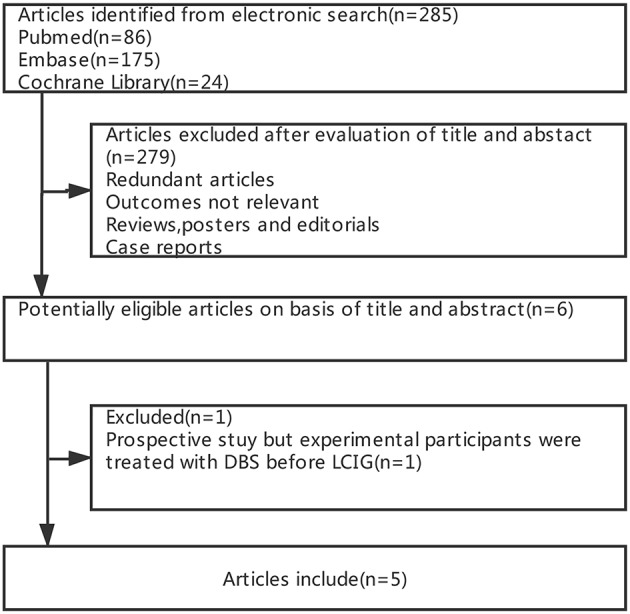
Process of study selection.

Figure 1 summarizes the process of study selection. Of 285 potentially relevant articles from electronic databases, 279 were excluded after screening the titles and abstracts (e.g., the content or outcomes of some studies were irrelevant, some studies were reviews or case reports, and some were exhibited in the form of conference abstracts). Furthermore, among the remaining five articles, we excluded one article (14) as the participants were treated with subthalamic nucleus deep brain stimulation (STN-DBS) before LCIG, which was not in accordance with our inclusion criteria. Therefore, we included five cohort trials (15–19) of 257 patients in the meta-analysis (Table 2).

**Table 2:** Characteristics of included studies.

**Items**	**Group**	**References**				
		**Merola et al. (17)**	**Merola et al. (15)**	**Elia et al. (16)**	Dafsari et al. (19)^*^	**Valldeoriola et al. (18)**
**Participants**						
Sample size	LCIG group	20	20	10	33 (25^a^)	11
	STN-DBS group	20	20	10	101 (25^a^)	12
Age (years)	LCIG group	64.60 ± 6.99	69.00 ± 5.90	68.40 ± 1.60	64.40 ± 8.30	64 (59, 72)^c^
	STN-DBS group	64.05 ± 5.76	66.60 ± 2.50	55.20 ± 2.20	64.10 ± 8.30	57 (51, 63)^c^
Male (%)	k	NR	13 (65)	4 (40)	14 (56)	8 (72.7)
	STN-DBS group	NR	16 (80)	6 (60)	9 (36)	11 (91.7)
Disease duration (years)	LCIG group	13.75 ± 2.57	13.90 ± 4.50	14.00 ± 1.70	13.80 ± 4.90	14.5
	STN-DBS group	13.80 ± 3.09	16.40 ± 4.30	15.60 ± 1.80	12.70 ± 4.20	13.0
Hohen-Yahr stage	LCIG group	2.39 ± 0.74	NR	2–3	3.0 (3.0, 4.0)^c^	2.5 (2.5, 2.5)^c^
	STN-DBS group	2.26 ± 0.59	NR	2–3	3.0 (3.0, 3.5)^c^	2.3 (2.0, 2.5)^c^
Baseline LEDD (mg/day)	LCIG group	1272.0 ± 432.0	994.5 ± 268.0	NR	1472.2 ± 707.0	NR
	STN-DBS group	1383.0 ± 458.0	1220.0 ± 452.0	NR	1179.4 ± 580.2	NR
Outcome assessment		Activity of Daily Living Scale (ADL), OFF time, dyskinesia, motor severity, neuropsychological outcome, pharmacological therapies and stimulation parameters, adverse event;	UPDRS, neuropsychological and behavioral tests, adverse event;	Motor condition (includes UPDRS, hand tapping, time to best motor “on” state, number of “off” state epochs and of “on” state epochs), dyskinesia, adverse event;	PDQ-8 SI, UPDRS-III, UPDRS-IV, Hohen-Yahr stage, LEDD, nonmotor Symptoms Scale (NMSS), adverse event;	Cognitive assessment, mood and behavior assessment, fatigue assessment, motor evaluation, medication (L-dopa equivalent dose), adverse event;
Compared index for meta-analysis		UPDRS-III, UPDRS-IV, adverse event	UPDRS-III, UPDRS-IV, adverse event	UPDRS-III, UPDRS-IV, adverse event	UPDRS-III, UPDRS-IV, adverse event	Adverse event
Study design and follow-up duration (months)	LCIG group	Retrospective-cohort study; 61.80 (36–102)^b^	Retrospective-cohort study; 14.70 ± 7.60	Retrospective-cohort study; 13.80 ± 1.50	Prospective-cohort study; 6	Prospective-cohort study; 12
	STN-DBS group	Retrospective-cohort study; 60.96 (36–108)^b^	Retrospective-cohort study; 14.80 ± 3.30	Retrospective-cohort study; 21.90 ± 5.90	Prospective-cohort study;6	Prospective-cohort study; 12
Comment	Some participants (*n* = 20) of this study received OMT besides LCIG and DBS.		Some participants (*n* = 10) of this study received subcutaneous apomorphine besides LCIG and DBS.	Some participants (*n* = 39) of this study received subcutaneous apomorphine besides LCIG and DBS.	Some participants (*n* = 20) of this study received OMT besides LCIG and DBS.	

*LCIG, Levodopa/carbidopa intestinal gel infusion; STN-DBS, Subthalamic nucleus deep brain stimulation; LEDD, Levodopa Equivalent Daily Dose; NR, Not reported; UPDRS, Unified Parkinson Disease Rating Scale; OMT, Oral medical therapy*.

a *This is a matched cohort which is from an original cohort by a method called propensity score matching*.

b *Results are shown as the mean (range)*.

c *Data are represented as median [25th, 75th percentiles] for the variables*.

* *The data of clinical and demographic characteristics of this trial was caculated from matched cohort*.

### Study Characteristics

The clinical and demographic characteristics of included studies are summarized in Table 2. All studies presented results for the patients who received STN-DBS or LCIG. Additionally, four trials (16–19) included oral medical therapy (OMT) and/or subcutaneous apomorphine infusion. Although the clinical assessment tools varied among the studies, they all evaluated motor function, dyskinesia and adverse events or complications. Moreover, two studies (15, 17) recorded neuropsychological outcomes; one (19) assessed non-motor symptoms scale (NMSS); and the other one (18) evaluated cognition, mood, behavior, and fatigue.

The ages of patients in most of these studies were similar except for two trials (16, 18); the patients who received STN-DBS were significantly younger than those in the LCIG groups. As for the sex of the patients, more men seemingly preferred to choose advanced treatments. The disease duration of patients was long enough, which represented poor response to oral medicine such as levodopa. Additionally, the Hohen-Yahr stages were almost equivalent among the groups, but one study (15) showed that the baseline LEDD was higher in the STN-DBS group.

### Methodological Quality

The results for quality assessment of included studies by NOS are listed in Table 3. All cohort trials selected patients who were somewhat representative of the average PD patients in their community, which fulfilled unified clinical and neuropsychological criteria. Additionally, PD patients in both cohorts were enrolled from the same disease centers in each study. As for ascertainment of intervention exposure, all trials used validated measures—such as dose conversion of levodopa—in the administration and criteria for surgical procedures and other interventions. All groups verified that any advanced treatment (e.g., DBS) was not present at the start of the study. Regarding comparability, three trials (17–19) managed to control for both age (gender) and disease characteristics, while the other two studies (15, 16) only controlled one factor. Ultimately, none of the studies lost any stars on the outcome subscale except for one (19). The follow-up duration of 6 months was short in this trial. As a result, researchers performed a cautious assessment and reduced possible bias as much as possible.

**Table 3:** Quality assessment of observational studies on advanced treatment of PD^a^.

**References**	**Selection 1**	**Selection 2**	**Selection 3**	**Selection 4**	**Comparability 1**	**Exposure 1 outcome 1**	**Exposure 2 outcome 2**	**Exposure 3 outcome 3**	**Total**
Merola et al. (17)	1	1	1	1	2	1	1	1	9
Merola et al. (15)	1	1	1	1	1	1	1	1	8
Elia et al. (16)	1	1	1	1	1	1	1	1	8
Dafsari et al. (19)	1	1	1	1	2	1	0	1	8
Valldeoriola et al. (18)	1	1	1	1	2	1	1	1	9

a *Numbers represent the stars allocated to each question (see Table 1 for guidance)*.

### Comparison of LCIG and STN-DBS

**Figure 2:**
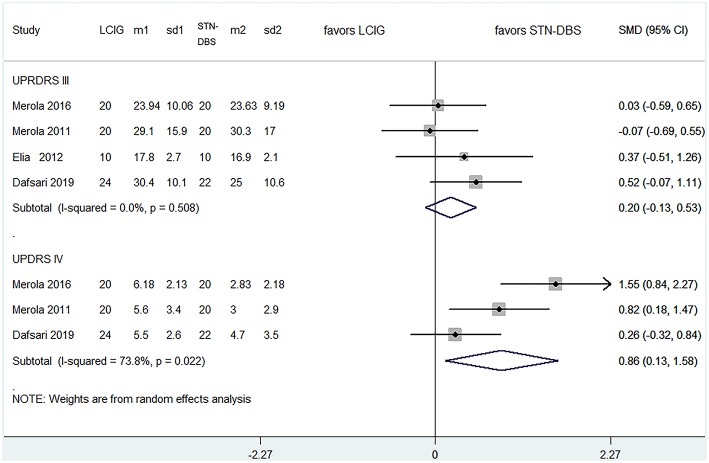
Comparisons between LCIG and STN-DBS for UPDRS III, UPDRS IV. LCIG, Levodopa/carbidopa intestinal gel infusion; STN-DBS, Subthalamic nucleus deep brain stimulation; m, mean; sd, standard error.

All studies investigated the comparison between LCIG and STN-DBS (Table 4), which fulfilled our inclusion criteria. As for motor function, there was no statistical difference among four trials (15–17, 19) for UPDRS-III: the pooled SMDs were 0.200 (95% CI −0.126–0.527, *P* = 0.230). Notably, the improvement of UPDRS-IV was more significant in STN-DBS groups: the pooled SMDs were 0.857 (95% CI 0.130–1.584, *P* = 0.021) for three trials (15, 17, 19) (Figure 2). However, the heterogeneity of this part was significant (*I*^2^ = 73.8%). Meta-regression was not available because of the limited number of comparable trials. So, we tried to analyze the possible source of heterogeneity by demographic and clinical characteristics of these studies (see Discussion section).

**Table 4:** Summary of the efficacy, heterogeneity, and publication bias.

**Outcomes**	**No of trials; No of comparisons**	**No of participants**	**SMD (95% CI) or RR (95%CI)**	**Test of SMD or RR (*P*-value)**	**Heterogeneity**		**Publication bias (*****P*****-value)**	
					***P*-value**	**I^**2**^ (%)**	**Begg's test**	**Egger's test**
**UPDRS**								
UPDRS-III	4 (15–17, 19); 4	146	0.200 (−0.126, 0.527)^*^	0.230	0.508	0.0	0.872	0.872
UPDRS-IV	3 (15–19); 3	126	0.857 (0.130, 1.584)^*^	0.021	0.022	73.8	0.051	0.051
**ADVERSE EVENT**								
Total adverse event	5 (15–19); 5	257	1.279 (0.983, 1.664)	0.067	0.521	0.0	0.193	0.193
Serious adverse event	4 (15–17, 19); 4	234	1.539 (0.664, 3.566)	0.315	0.967	0.0	0.154	0.154

*No, Number; SMD, Standard mean difference; RR, Relative Risk; CI, Confidence Interval; UPDRS, Unified Parkinson Disease Rating Scale*.

* *From random effects model*.

**Figure 3:**
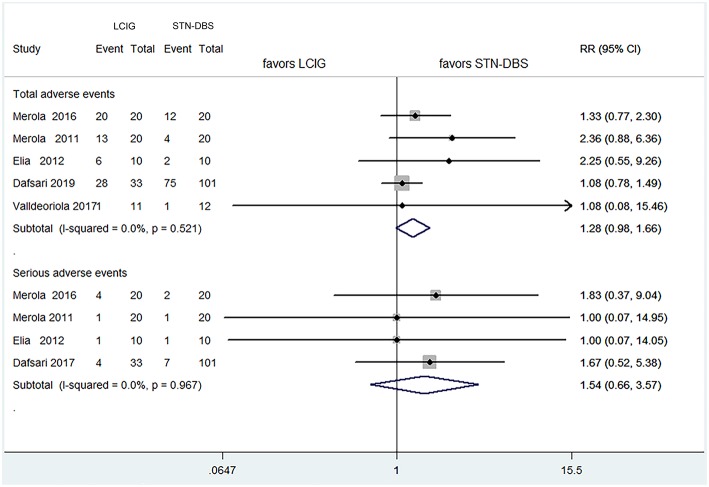
Comparisons between LCIG and STN-DBS for adverse events. LCIG, Levodopa/carbidopa intestinal gel infusion; STN-DBS, Subthalamic nucleus deep brain stimulation.

With regard to the comparisons for total adverse events and serious adverse events, the pooled RRs were 1.279 (95% CI 0.983–1.664, *P* = 0.067) for all trials, and 1.539 (0.664–3.566, *P* = 0.315) for four studies (15–17, 19), respectively (Figure 3). These results showed no statistical difference as that of UPRDS-III either.

Formal investigation using Begg's test and Egger's test did not detect significant publication bias among all comparisons (Table 4). Funnel plot for publication bias were showed in the (Supplementary Figure 1). Furthermore, some results which were unavailable in meta-analysis still demonstrated statistical differences among the studies. Three trials (15, 17, 19) found that the LEDD reductions were significant in STN-DBS groups. Additionally, a similar result was applied for the time required to reach the best “on” status, which refers to PD patients being able to move freely again (16). Notably, the other included study (18) used more complicated tests to assess the cognitive functions between groups, which suggests that the ability of learning and recall was significantly improved by LCIG compared with that of STN-DBS.

### Sensitivity Analyses

We carried out sensitivity analyses by removing any one trial in each endpoint. The results showed that the outcomes for all comparisons did not change after excluding any one study. The pooled SMDs or RRs without any one trial did change significantly (Supplementary Figure 2). It provided a robust balance for these endpoints. The Galbraith plot was used to spot identified studies as important sources of heterogeneity. For the comparisons in UPDRS-IV, we excluded trials that did not fall within two standard deviations of the z score by sensitivity analyses. Consequently, after excluding one trial (17), the comparison of UPDRS-IV showed the biggest change (pooled SMD 0.523, 95% CI −0.526–1.072) (Supplementary Table 4). As the number of included studies were small, we did not make a subgroup analysis. The reason of heterogeneity arising will be discussed in the Discussion section.

## Discussion

### Important Findings of This Study

The result of comparisons for UPDRS-III indicated that there were no significant differences in the improvement of motor function for advanced PD patients, whether they received LCIG or STN-DBS. Indeed, to our knowledge, current evidence based on direct comparisons between these therapeutic methods is limited. There are positive therapeutic effects of STN-DBS on the motor function of advanced PD patients, which have been validated by numerous clinical trials (20–23). Additionally, STN-DBS has been recommended by the International Parkinson and Movement Disorder Society (MDS) in a recent evidence-based medicine review (24). However, the introduction of LCIG to clinical application is relatively recent. Although there have been some LCIG-based clinical trials (8, 25–28) to confirm its value for advanced PD patients, the evidence reviewed by MDS was relatively insufficient (24). Our present review suggests that, in terms of improvement of motor function, LCIG has comparable advantage as STN-DBS compared with oral levodopa treatment.

With regards to UPDRS-IV, the result demonstrated that STN-DBS has shown superior efficacy on improvement of dyskinesia or motor fluctuation, compared with LCIG. To the best of our knowledge, there are currently no similar findings based on direct comparisons between these advanced treatments. Regarding the possible mechanism for our findings, we hypothesize that DBS has various effects on the cortico-basal ganglia loop, breaks up mutual signals from the stimulated nuclei, and disrupts abnormal informational flow through the cortico-basal ganglia loop in pathological conditions (29). Similarly, LCIG has the capacity to influence both pharmacokinetic and pharmacodynamic factors of levodopa, which may be related to improvement of troublesome dyskinesia (30, 31). However, the pathophysiological mechanism of levodopa-induced dyskinesias (LID) is complex: it comprises a combination of nigrostriatal degeneration, pulsatile dopaminergic stimulation, and synaptic remodeling (32). Compared with LCIG, the effect of improvement on LID by STN-DBS is mostly dependent on a reduction of levodopa dosage (33). And this conclusion is coincident with the result in our review: the LEDD reductions were significant in STN-DBS groups in the three included studies (15, 17, 19). What's more, some previous studies indicated that STN-DBS might also target some locations in the cortico-basal ganglia loop to provide better control of dyskinesia (34). Therefore, it can more effectively improve LID with respect to its putative pathophysiological mechanism. Nevertheless, the conclusion of this comparison should be treated cautiously. Because the pooled results had a moderate heterogeneity (*I*^2^ = 73.8%). And we found out one trial (17) as the possible source of heterogeneity by sensitivity analyses. As the number of included studies was small, it is difficult to make a meta-regression or a subgroup analysis. By comparing the demographic and clinical characteristics of these studies, we found the biggest difference was the follow-up duration. So, we deduced that a possible significant source of the heterogeneity was from the follow-up duration of these trials. In fact, a better improvement is often associated with a longer therapeutic duration. As mentioned above, since STN-DBS has relative superiority than LCIG on improvement of dyskinesia theoretically, it is not strange that the study (17) which caused the moderate heterogeneity showed the best performance of STN-DBS for UDPRS-IV (Figure 3). But more well-designed trials are needed to confirm this conclusion.

As far as adverse events were concerned, the results suggested that the incidence of adverse events for LCIG were similar to STN-DBS. Although the procedure-related complications of LCIG and STN-DBS were frequent, the comparison for serious adverse events showed that adverse events related to both of them were occasionally life threatening. This finding is coincident with most previous investigations (6, 8, 30, 35, 36). Moreover, an observation from the safety data from four related studies was that most adverse events had been resolved within the first 4 weeks after percutaneous enteral gastrostomy by LCIG (37). Therefore, this review suggests that despite the high rates of adverse events, the safety and tolerance of LCIG and STN-DBS for advanced PD patients are generally acceptable. Nevertheless, it is important that multi-disciplinary teams are supported by movement-disorder specialists, gastrointestinal experts, routine return visits, regular care of tubing, and relevant education of patients (38).

As for some of the data excluded by meta-analysis in our review, we found some interesting differences in treating some non-motor symptoms. Firstly, two studies (18, 19) suggested that the improvement of cognitive functions—especially for the ability of learning and recall—was more significant in the LCIG groups, compared with that of the STN-DBS groups. However, to our knowledge, relevant research on the effects of LCIG and STN-DBS on cognition is limited at present. By reviewing the electronic database, only some case reports (18, 39) have shown remarkable improvement of cognitive functions with LCIG. Similarly, the effects of STN-DBS on cognitive function remain controversial. Most studies have suggested that STN-DBS is relatively safe with respect to its impact on cognition, while other studies have considered that STN-DBS may cause subtle declines in intelligence, memory, verbal fluency, and executive function (40). Regardless, the reasons for the differences in impacts on improvement of cognitive function between LCIG and STN-DBS are still unclear. Dopaminergic drugs, especially levodopa, have some positive influences on cognitive function (18). We consider that a possible mechanism for LCIG efficacy is that it can ensure continuous stimulation of dopaminergic transmission, avoiding pulsatile dopaminergic stimulation by oral levodopa, resulting in a relative stability of dopaminergic signaling (41). On the contrary, STN-DBS cannot directly change the level of stimulation of dopaminergic transmission. Although it may show some extent of improvement of some aspects of cognition (42), the therapeutic value for this may be relatively small than for LCIG. Next, with respect to sleep/fatigue, urinary symptoms and sexual function, one included trial (19) demonstrated that STN-DBS had more beneficial effects on these symptoms. And the result has confirmed some previous STN-DBS related studies (43–45). However, as related evidence is still limited at present, it is necessary to carry out more large cohorts or randomized trials to corroborate this conclusion.

### Limitations of the Study

There are several considerable limitations in our review: Firstly, sample sizes of the included trials were relatively small, which may increase the risk of enlarging sampling errors. Secondly, the follow-up duration of the patients varied among the involved trials, which was possibly an important source of heterogeneity for the comparison in the UPDRS-IV group. Additionally, a long enough period of follow-up is a key index to assess long-term therapeutic effects of these advanced treatments. Thirdly, none of the reports included cost-effective analysis of advanced therapies, which is an underestimated aspect for the relevant research. As both LCIG and STN-DBS are relatively expensive (5, 46, 47), assessment of the cost-effective analysis is important. Finally, we found little focus on other types of DBS stimulating different nuclei, such as the internal globus pallidus (GPi) nucleus, which may undervalue the importance of DBS in some aspects of treating advanced PD patients.

### Implications for Clinical Practice

The most consistent evidence was that LCIG had shown equivalent effects compared to STN-DBS on improvement of motor function for advanced PD patients. Consequently, it would be helpful to provide some rational clinical advice for patients who are not suitable for STN-DBS. Additionally, some data in our review suggested that STN-DBS may have priority over LCIG for patients suffering from more severe dyskinesia or motor fluctuation. Likewise, two studies (18, 19) in our review found that LCIG may have more positive impact than STN-DBS on the improvement of cognitive function, especially for learning and memory. Therefore, for PD patients with troublesome dyskinesia or motor fluctuation, STN-DBS would be an optimal option compared with LCIG. On the contrary, regarding patients with cognitive impairment or dementia, it might be recommended to choose LCIG rather than STN-DBS. However, the relevant evidence of both results is currently not persuasive. As a result, it is essential to carry out more clinical trials, especially for large cohorts or RCTs.

### Directions for Future Research

In future research, a better evidence-based review is needed to extend the recommendations for clinical practice on advanced therapies for advanced PD. All of the included studies in our review were small cohort trials, and the levels of evidence were relatively low. Therefore, more RCTs and larger cohorts with better methodological qualities are required. Actually, we have saved each search item with time label (see the Supplementary Table 1) in our search strategy, and set email alerts for those electronic databases. Once there are new valuable results, we will add them to our future work. Furthermore, it is essentially important to utilize more reliable and sensitive measures to evaluate various outcomes of advanced PD patients, particularly for cognitive functions and neuropsychological tests.

## Conclusion

LCIG has comparable effects to STN-DBS on motor function for advanced PD, and their tolerability is acceptable. Although some trials suggested STN-DBS and LCIG had more beneficial effects on motor complications and cognitions, respectively, it is still advisable that clinical physicians make individualized choices based on individual condition of each patient. And larger, well-designed trials are needed to test the comparability of LCIG and STN-DBS in the future.

## Data Availability

All datasets for this study are included in the manuscript and the Supplementary Files.

## Author Contributions

XL conceived and designed the study. XL and YB collected and analyzed the data. The manuscript was drafted and reviewed by all authors. All authors took responsibility for the accuracy and integrity of the data reported and made the decision to submit the manuscript for publication.

### Conflict of Interest Statement

The authors declare that the research was conducted in the absence of any commercial or financial relationships that could be construed as a potential conflict of interest.
