# Supplementary material for: Comparison Between Levodopa-Carbidopa Intestinal Gel Infusion and Subthalamic Nucleus Deep-Brain Stimulation for Advanced Parkinson's Disease: A Systematic Review and Meta-Analysis
Source: Front Neurol. 2019 Aug 27;10:934. doi: 10.3389/fneur.2019.00934 (PMC6718716; doi:10.3389/fneur.2019.00934)
Supplement: Supplementary file 4 [file Table_4.DOCX]

S-Table 4. Results for Sensitivity Analyses

UPDRS-III:

Study SMD [95% Conf. Interval] % Weight

Merola 2016 0.032 -0.588 0.652 27.79

Merola 2011 -0.073 -0.693 0.547 27.77

Elia 2012 0.372 -0.513 1.257 13.63

Dafsari 2019 0.522 -0.067 1.111 30.81

D+L pooled SMD 0.200 -0.126 0.527 100.00

Heterogeneity chi-squared = 2.32 (d.f. = 3) p = 0.508

I-squared (variation in SMD attributable to heterogeneity) = 0.0%

Estimate of between-study variance Tau-squared = 0.0000

Test of SMD=0: z= 1.20 p = 0.230

Study omitted Estimate [95% Conf. Interval]

Merola 2016 .26496484 -.11954332 .649473

Merola 2011 .30532062 -.07914591 .68978715

Elia 2012 .17221754 -.19269258 .53712766

Dafsari 2019 .05697629 -.33582408 .44977665

Combined .20027634 -.12646881 .52702149

UPDRS-IV:

Study SMD [95% Conf. Interval] % Weight

Merola 2016 1.554 0.843 2.266 31.57

Merola 2011 0.823 0.176 1.470 33.32

Dafsari 2019 0.261 -0.320 0.842 35.11

D+L pooled SMD 0.857 0.130 1.584 100.00

Heterogeneity chi-squared = 7.62 (d.f. = 2) p = 0.022

I-squared (variation in SMD attributable to heterogeneity) = 73.8%

Estimate of between-study variance Tau-squared = 0.3039

Test of SMD=0 : z= 2.31 p = 0.021

Study omitted Estimate [95% Conf. Interval]

Merola 2016 .52330105 -.02583756 1.0724397

Merola 2011 .89084132 -.37604818 2.1577308

Dafsari 2019 1.172975 .4566657 1.8892843

Combined .85661854 .12969234 1.5835448

Total adverse events:

Study RR [95% Conf. Interval] % Weight

Merola (2016) 1.333 0.774 2.298 22.29

Merola (2011) 2.364 0.879 6.359 7.74

Elia (2012) 2.250 0.547 9.257 3.82

Dafsari (2019) 1.077 0.781 1.486 64.54

Valldeoriola (2017) 1.083 0.076 15.463 1.60

M-H pooled RR 1.279 0.983 1.664 100.00

Heterogeneity chi-squared = 3.22 (d.f. = 4) p = 0.521

I-squared (variation in RR attributable to heterogeneity) = 0.0%

Test of RR=1 : z= 1.83 p = 0.067

Study omitted Estimate [95% Conf. Interval]

Merola (2016) 1.2631357 .93521701 1.7060339

Merola (2011) 1.1877355 .9051127 1.5586076

Elia (2012) 1.240197 .94931841 1.6202031

Dafsari (2019) 1.645779 1.0486269 2.5829858

Valldeoriola (2017) 1.2819705 .98443711 1.6694294

Combined 1.2787826 .98293982 1.6636674

Serious adverse events:

Study RR [95% Conf. Interval] % Weight

Merola (2016) 1.833 0.372 9.042 27.25

Merola (2011) 1.000 0.067 14.954 13.06

Elia (2012) 1.000 0.071 14.053 13.06

Dafsari (2017) 1.668 0.517 5.376 46.64

M-H pooled RR 1.539 0.664 3.566 100.00

Heterogeneity chi-squared = 0.26 (d.f. = 3) p = 0.967

I-squared (variation in RR attributable to heterogeneity) = 0.0%

Test of RR=1: z= 1.00 p = 0.315

Study omitted Estimate [95% Conf. Interval]

Merola (2016) 1.4282178 .53073599 3.8433537

Merola (2011) 1.6194786 .66720588 3.930887

Elia (2012) 1.6194786 .66546851 3.9411495

Dafsari (2017) 1.4255319 .42677214 4.7616539

Combined 1.5386001 .66378354 3.5663586
