# Supplementary figures and images for: Comparison Between Levodopa-Carbidopa Intestinal Gel Infusion and Subthalamic Nucleus Deep-Brain Stimulation for Advanced Parkinson's Disease: A Systematic Review and Meta-Analysis
Source: Front Neurol. 2019 Aug 27;10:934. doi: 10.3389/fneur.2019.00934 (PMC6718716; doi:10.3389/fneur.2019.00934)

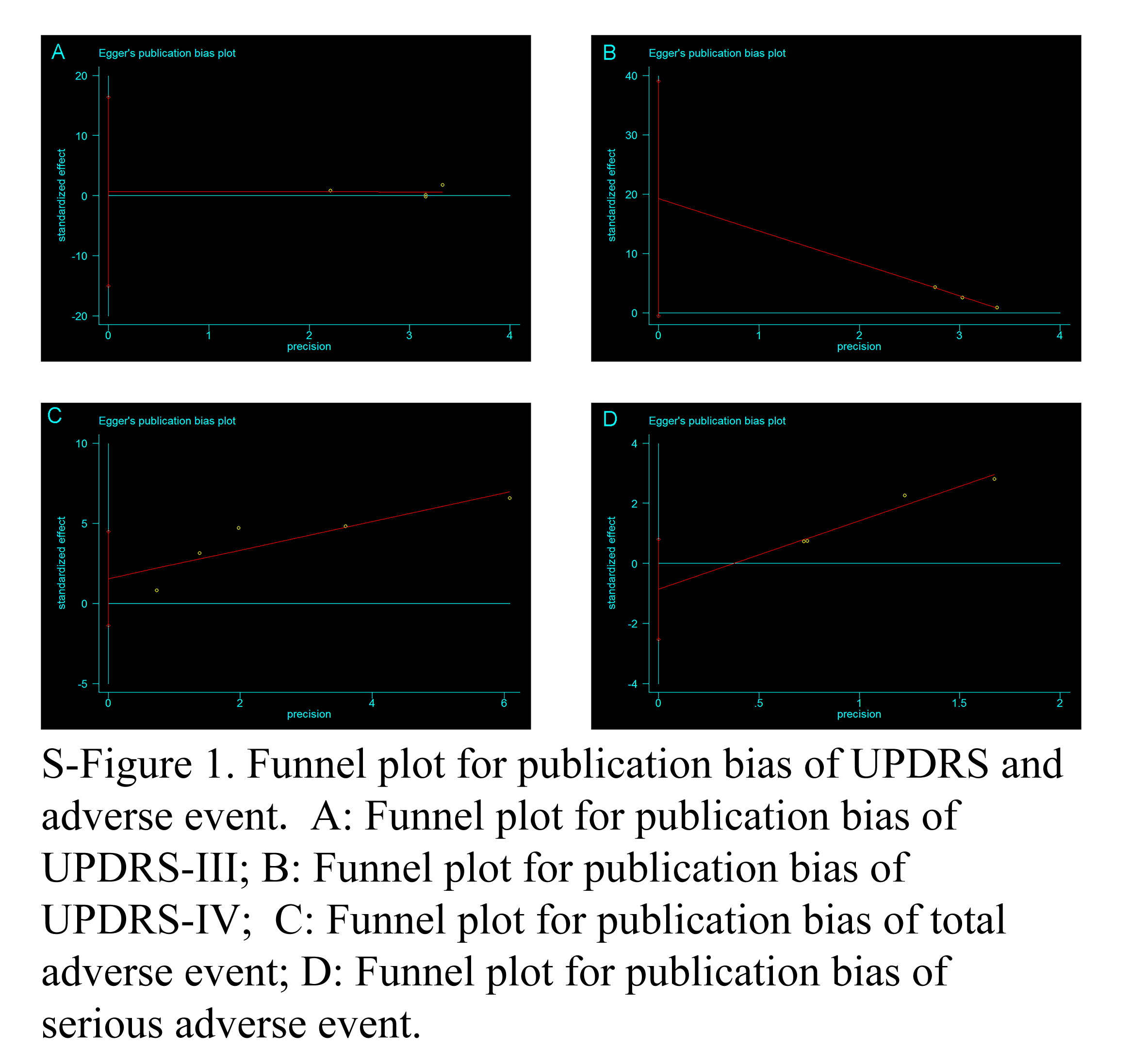

Supplement: Supplementary file 5 [file Image_1.TIF]

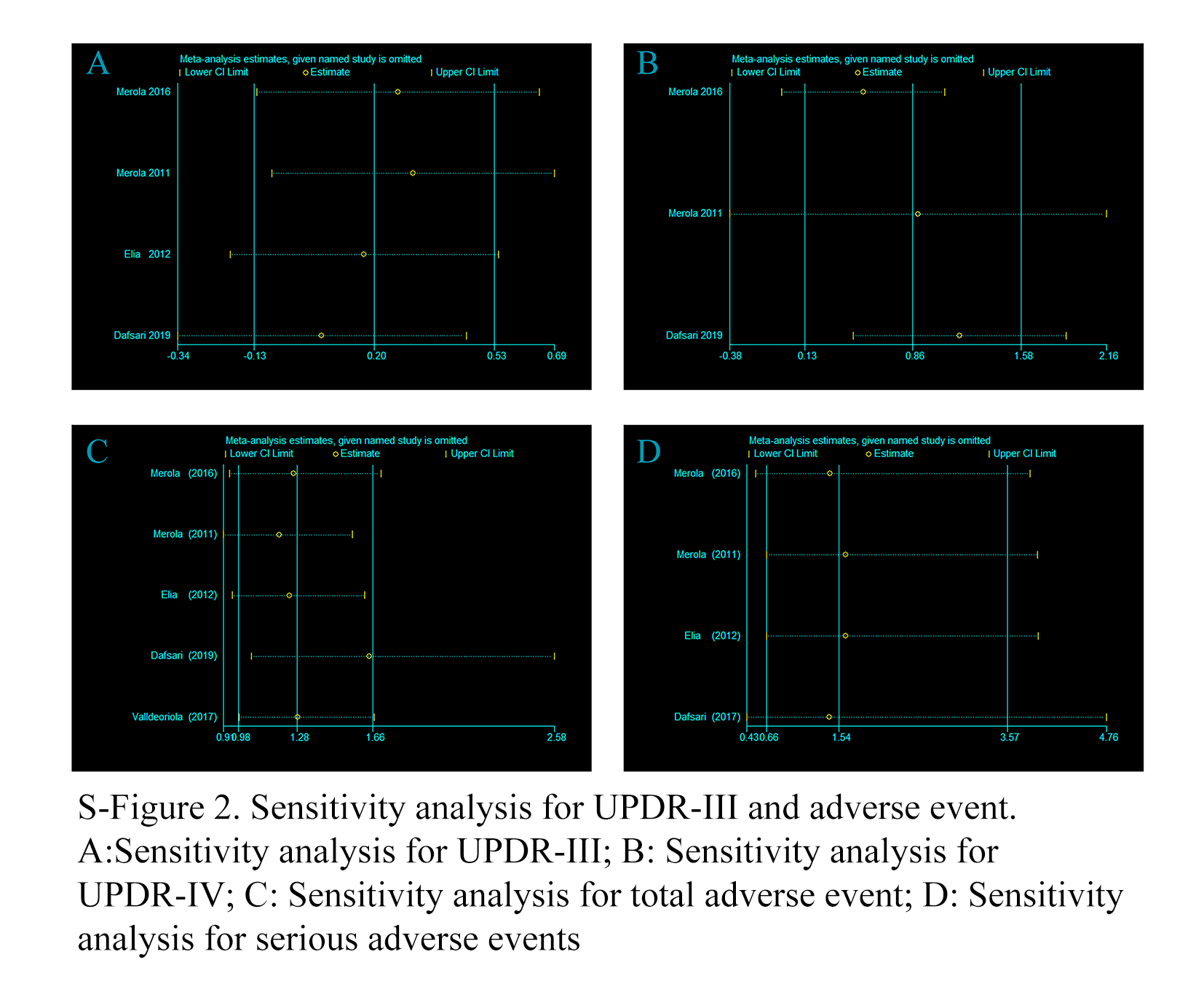

Supplement: Supplementary file 6 [file Image_2.TIF]
